# Factors Affecting Daily Functioning in Turkish Patients with Obstructive Sleep Apnea

**DOI:** 10.3390/medicina60101652

**Published:** 2024-10-09

**Authors:** Sengul Cangur, Ege Gulec Balbay, Terri E. Weaver

**Affiliations:** 1 Department of Biostatistics and Medical Informatics, Faculty of Medicine, Duzce University, 81620 Duzce, Turkey; 2 Department of Chest Diseases, Faculty of Medicine, Duzce University, 81620 Duzce, Turkey; egegulecbalbay@gmail.com; 3 Department of Biobehavioral Nursing Science, College of Nursing, University of Illinois Chicago, Chicago, IL 60612, USA; penntw@yahoo.com; 4 School of Nursing, University of Pennsylvania, Philadelphia, PA 19104, USA

**Keywords:** obstructive sleep apnea, daily functioning, functional outcomes, daytime sleepiness, depression, anxious, quality of life

## Abstract

*Background and Objectives:* This study aims to examine the factors affecting the daily functioning of patients with obstructive sleep apnea (OSA). *Materials and Methods:* In addition to the polysomnography records of 361 patients, participants completed the Turkish FOSQ-10 (Functional Outcomes of Sleep-10), Medical Outcome Survey Short Form-12, Epworth Sleepiness Scale (ESS), Beck Depression Inventory (BDI), and Beck Anxiety Inventory (BAI). First, the psychometrics properties of the Turkish FOSQ-10 were evaluated. Then, factors affecting daily functioning were examined through univariate and multivariate analyses. *Results:* Of all participants, 68.7% (*n* = 248) were male, and the average age was 47.94 ± 11.08. According to the OSA category, 23% *(n* = 83) were mild, 22.7% (*n* = 82) were moderate, 45.2% (*n* = 163) were severe, and 9.1% *(n* = 33) were OSA negative. The Turkish FOSQ-10 was found to be a valid and reliable scale through validity and reliability analyses. The moderate and severe OSA patients had different FOSQ-10 Total scores compared to the negative OSA group. Daily functioning was positively associated with overall quality of life while inversely associated with depression, being anxious, and daytime sleepiness in OSA patients. In a multiple regression model, BDI, mental component summary-12, physical component summary-12, and ESS scores were significantly related to the FOSQ-10 Total score in OSA patients (*p* < 0.05). *Conclusions:* The daily functioning of moderate and severe OSA patients was worse than that of the negative OSA group. Depression, quality of life, and daytime sleepiness were simultaneously important variables associated with daily functioning in OSA patients.

## 1. Introduction

OSA (obstructive sleep apnea) is characterized by recurrent episodes of complete (apnea) or partial (hypopnea) repetitive upper airway obstruction, frequently accompanied by a decrease in blood oxygen saturation [1]. According to the most comprehensive OSA prevalence study in 2019, which included 16 countries, the global prevalence of OSA in the global adult population aged 30–69 is approximately 25% [2]. The most common symptoms are daytime sleepiness, intense snoring, respiratory pauses, and choking during sleep. The daily functioning of patients with OSA is influenced by numerous factors encompassing physiological, psychological, and environmental areas. Physiological factors, such as the severity of upper airway obstruction, fragmentation of sleep architecture, and the degree of nocturnal hypoxemia, as well as sleep duration and efficiency, can significantly impact the quality of sleep and subsequent daytime functioning [3]. Additionally, comorbidities, including cardiovascular disease, metabolic syndromes, and psychiatric disorders, as well as motor vehicle crashes, contribute to the complexity of OSA management and further exacerbate functional impairments [4,5]. Psychological factors, such as anxiety, depression, and cognitive dysfunction, not only influence sleep patterns but also compliance with continuous positive airway pressure (CPAP) therapy, a mainstay treatment for OSA [6]. Understanding and addressing these complex factors are essential for optimizing therapeutic treatments and improving daily functioning and health-related quality of life in patients with OSA. Moreover, it is imperative to assess and follow up on the daily functioning and overall health-related quality of life in this population. Employing tools such as the Functional Outcomes of Sleep Questionnaire (FOSQ), a measure of the impact of sleepiness on daily functioning, or its short version, FOSQ-10, facilitates the practical evaluation of the effects of excessive daytime sleepiness (EDS) on an individual’s ability to engage in daily activities and perform certain functions, as well as to sustain social relationships [7,8].

Some researchers have found that untreated severe OSA patients exhibit diminished quality of life [9] and daily functioning [10], alongside elevated levels of depression and anxiety [5,11]. However, these results could not be obtained in some studies where these features were examined according to OSA severity [12,13,14,15,16].

The aim of this study is to examine the factors affecting daily functioning in patients with OSA. Since the FOSQ-10 has not been translated into Turkish, the translation and associated psychometric properties of the scale will also be examined in this study.

## 2. Material and Methods

### 2.1. Study Design

This study has a methodological and cross-sectional design.

### 2.2. Study Group

Between 15 June 2021 and 15 June 2022, 361 patients over the age of 18 without cognitive impairment, who underwent polysomnography (PSG) with a pre-diagnosis of OSA in Duzce University Hospital Sleep Laboratory, were included in the study. Ethics committee approval was obtained for this study from the ethics committee of Duzce University in Turkey (decision number: 2021/142).

### 2.3. Study Layout

During the diagnosis stage, a 14-channel video-recorded digital polysomnography device (Philips Respironics Model: Alice-6 PSG, Germany) was used for sleep monitoring. The polysomnographic record includes 2-channel electroencephalography (EEG), 2-channel electrooculography (EOG), submental electromyography (EMG), nasal flow, thoracic and abdominal movement, pulse and oxygen saturation, anterior tibialis EMG, electrocardiography (ECG), and snoring sound. Those with an apnea-hypopnea index (AHI) = 5–14.9 were considered mild, those between 15–29.9 were considered moderate, and those 30 and over were considered severe sleep apnea [1]. During the first visit, the Turkish FOSQ-10, Medical Outcome Survey Short Form-12 (SF-12), Epworth Sleepiness Scale (ESS), and Beck Depression and Anxiety Inventories (BDI, BAI) were manually administered to all individuals in the study by a somnologist in accordance with the instrument scoring instructions and the American Academy of Sleep Medicine (AASM) 2014 guidelines.

### 2.4. Tools

Epworth Sleepiness Scale (ESS): The ESS is an eight-item scale developed by Johns [17] to assess daytime sleepiness. Utilizing a four-point scoring system (ranging from 0 to 3), total scores on the scale range from 0 to 24, with higher values denoting increased daytime sleepiness. An ESS score of >10 indicates pathological sleepiness or EDS. Agargun et al. [18] conducted a Turkish validity and reliability study of the scale.

Functional Outcomes of Sleep Questionnaire-10 (FOSQ-10): Chasens et al. [8] developed the FOSQ-10 by identifying items from each subscale of the original FOSQ, which included 30 items categorized into five factors, based on their substantial effect sizes observed before and after treatment. This tool is used to determine whether patients have difficulty performing certain activities due to sleepiness or fatigue [7]. In this study, the psychometric properties of the short form were initially assessed, as there was no prior investigation establishing its validity and reliability within the Turkish population. 

With a four-point scoring system (1–4), the 5 subscales of the 10 items of the tool are as follows: General Productivity (Item 1: Concentrating, Item 2: Remembering), Activity Level (Item 6: Relationships affected, Item 8: Activity in morning, Item 9: Activity in evening), Vigilance (Item 3: Driving short distance, Item 4: Driving long distance, Item 7: Watching movies), Social Outcomes (Item 5: Visit in their home), and Sexual Relationship (Item 10: Desire intimacy/sex). In order to obtain the Total score, the average weighted item score was calculated for the subscales containing more than one item. Thus, the deterioration of the score caused by missing responses was prevented. The total score was obtained by calculating the mean of the subscales scores and multiplying by 5, regardless of the number of subscales. The total score ranges from 5 to 20, with higher scores indicating better functional status. Chasens et al. [8] found the Cronbach’s alpha of the tool to be 0.87. 

The standard forward–backward method was used to translate the English version of FOSQ-10 into Turkish. First, the original tool was translated into Turkish by two independent physicians and a translator. Then, three translations were converted into a single tool by an independent bilingual physician. Finally, the Turkish form was translated into English by two independent translators. 

Medical Outcome Survey Short Form-12 (SF-12): Ware et al. [19] reduced the number of items in the SF-36 and developed the SF-12. The SF-12 is a general quality of life tool that measures eight important dimensions of health: physical functioning, role limits—physical, bodily pain, general health, vitality, social functioning, role limits—emotional, and mental health. Items related to role limits—physical and role limits—emotional were answered as dichotomies (yes or no), while other items had Likert options ranging from 3 to 6. While the physical component summary-12 (PCS-12) score is obtained from the subscales of physical functioning, role limits—physical, bodily pain, and general health, the mental component summary-12 (MCS-12) score is obtained from the subscales of vitality, social functioning, role limits—emotional, mental health. The approach of Ware et al. [19] was taken into account, with a mean of 50 and a standard deviation of 10, in order to obtain interculturally comparable and interpretable scores for each subscale score calculation. Both PCS-12 and MCS-12 range from 0 to 100, with higher scores representing better health [19]. The Turkish validity and reliability study of the SF-12 was conducted by Soylu and Kutuk [20].

Beck Depression Inventory (BDI): This tool was developed by Beck et al. (1961) to measure the severity of depression and monitor changes. The BDI includes 21 self-assessment items. Each item is scored between 0 and 3, and the total score ranges from 0 to 63. A score of 0–9 indicates no depression, a score of 10–16 indicates mild depression, a score of 17–29 indicates moderate depression, and a score of 30 and above indicates severe depression [21]. The Turkish validity and reliability study was performed by Hisli [22].

Beck Anxiety Inventory (BAI): The scale was developed by Beck et al. [23] to measure the frequency and severity of anxiety-related symptoms. The BAI includes 21 self-assessment items. Each item is scored between 0 and 3, and the total score ranges from 0 to 63. A score of 0–7 indicates no anxious, a score of 8–15 indicates mild anxious, a score of 16–25 indicates moderate anxious, and a score of 26 and above indicates severe anxious. The Turkish validity and reliability study was conducted by Ulusoy et al. [24].

### 2.5. Statistical Analysis

Appropriate descriptive statistics were calculated according to the type of data and analysis. Univariate normality was evaluated with the Shapiro–Wilk test, as well as skewness and kurtosis coefficients, while the assumption of homogeneity of group variances was examined with the Levene test. One-way ANOVA (post-hoc Gabriel and Games–Howel tests) and Kruskal–Wallis (post-hoc Dunn test) were performed for group comparisons of quantitative variables. The relationships between quantitative variables were examined with Pearson and Spearman correlation tests. The Pearson Chi-square test was used to examine the relationship between categorical variables. Linearity, autocorrelation, and multicollinearity, which are the other important assumptions of multiple regression analysis apart from normality, were examined with scatter plot, Durbin–Watson, and variance inflation factor (VIF) approaches, respectively. A multiple regression analysis with a forward variable selection approach was applied to simultaneously examine each factor affecting the FOSQ-10 Total score.

The face, content, concurrent, and construct validities of the Turkish FOSQ-10 were examined. Cronbach’s alpha, McDonald’s omega, and test-retest coefficients were calculated for the reliability analysis of the tool. The multivariate normality and multicollinearity assumptions were assessed using Mardia’s multivariate normality test and the VIF approach, respectively. The impact score of each item in the scale was calculated for face validity. For content validity, the item-CVI (content validity index), overall average CVI, and universal CVI coefficients were calculated. Kendall’s W concordance coefficient and intra-class correlation (ICC) were calculated to assess agreement among the professional team for the suitability of the translation form. For concurrent validity, the relationships between the tools were determined using the Pearson correlation coefficient. To examine construct validity, the factor structure was first decided by MinRes exploratory factor analysis (EFA) (varimax rotation). At this stage, sampling adequacy was examined with the Kaiser–Meyer–Olkin test, and Bartlett’ sphericity test was used to determine the suitability of the data entry matrix. To test the validity of the determined factor structure, confirmatory factor analysis (CFA), which is a part of structural equation modeling, was applied using the maximum likelihood estimation technique. The model fitness was evaluated using fit indices such as (χ^2^/df), root mean square error of approximation (RMSEA), comparative fit index (CFI), goodness-of-fit index (GFI), adjusted goodness-of-fit index (AGFI), standardized root mean square residual (SRMR), and normed fit index (NFI). The SPSS v.22, LISREL 8.54, and RStudio 2023.06.0 programs, along with special macros, were used for statistical analyses. A *p* < 0.05 was considered statistically significant.

## 3. Results

### 3.1. Demographic and Clinical Characteristics of the Sample

Of the 361 people included in the study, 90.9% (*n* = 328) were diagnosed with OSA, while 9.1% (*n* = 33) were not diagnosed with OSA or any other sleep disorder but had symptoms such as snoring, daytime sleepiness. Of the individuals in the study, 31.3% (*n* = 113) were female, 68.7% (*n* = 248) were male, and the average age was 47.94 ± 11.08. According to OSA severity, 23% (*n* = 83) were mild, 22.7% (*n* = 82) were moderate, and 45.2% (*n* = 163) were severe, while 9.1% (*n* = 33) were OSA negative. Individuals’ sociodemographic characteristics, PSG measurements, and tool scores according to the OSA category, such as mild, moderate, severe, or negative OSA, are given in Table 1. The OSA category were homogeneous in terms of age, gender, and the rates of chronic diseases (*p* > 0.05). The severe OSA patients had significantly higher body mass index than those in the other groups (*p* < 0.001 for mild, negative OSA; *p* = 0.005 for moderate). The ESS scores of moderate (*p* = 0.041) and severe (*p* = 0.022) OSA patients were higher than those of the negative OSA group. The severe OSA patients had significantly higher PSG measures, such as AHI, and percentage of total sleep time with oxygen saturation < 90 (T90), than the other groups (*p* < 0.001). The moderate OSA patients had higher AHI (*p* < 0.001) and T90 (*p* = 0.008, *p* < 0.001) than the mild and negative OSA groups. The mild OSA group had higher AHI (*p* = 0.017) and T90 (*p* = 0.031) than the negative OSA group. Total sleep duration (TST) and sleep efficiency (SE) did not differ according to the OSA category (*p* > 0.05). The REM (rapid eye movement) sleep time of severe OSA patients was lower than that of the mild OSA (*p* = 0.030) and negative OSA (*p* = 0.020) groups. The percentage of TST in sleep stage N1 (nonrapid eye movement) of the severe OSA patients was higher than that of the mild (*p* = 0.031). The percentage of TST in sleep stage N2 of the severe OSA group was higher than that of the mild (*p* = 0.001), moderate (*p* < 0.001), and negative OSA (*p* < 0.001) groups, while the percentage of TST in sleep stage N3 of the severe OSA group was lower than that of the mild (*p* < 0.001), moderate (*p* = 0.001), and negative OSA (*p* = 0.030) groups.

### 3.2. The Psychometrics Properties of the FOSQ-10

The Turkish validity and reliability analysis results of the FOSQ-10 are summarized in Table 2. Table 2 also lists the criteria for evaluating the validity and reliability results of the tool. When the results of the tool were compared with these criteria, the instrument was found to be valid and reliable [25,26,27,28,29,30,31].

### 3.3. The Levels of Daily Functioning, Quality of Life, Depression, and Anxiety According to OSA Category

The FOSQ-10 Total, Activity Level, and Social Outcomes subscale scores were different according to the OSA category. The FOSQ-10 Total (*p* = 0.003, *p* = 0.037), Activity Level (*p* = 0.001, *p* = 0.033), and Social Outcomes (*p* = 0.001, *p* = 0.029) subscale scores were significantly lower in moderate and severe OSA patients compared to the negative OSA group. Moreover, the Social Outcomes subscale score of the FOSQ-10 was significantly lower in mild OSA patients compared to the negative OSA group (*p* = 0.017). The BDI and BAI scores of severe OSA patients were higher than those of mild (*p* = 0.001, *p* = 0.003) and negative OSA (*p* = 0.002, *p* < 0.001) groups. Additionally, the BAI score of moderate OSA patients was higher than that of the negative OSA group (*p* = 0.011). However, there were no significant differences between OSA categories in terms of General Productivity, Vigilance, and Sexual Relationship subscale scores of the FOSQ-10, and the PCS-12 and MCS-12 subscale scores of the SF-12 (*p* > 0.05, Table 3).

### 3.4. The Relationships between Daily Functioning and Participant Characteristics, PSG Measures, Quality of Life, Depression, and Anxiety

Table 4 shows the correlations between daily functioning and PSG measures, medical outcomes, depression, and anxiety. While a moderate negative correlation was found between the ESS and FOSQ-10 scores in OSA patients (*p* < 0.001), the AHI, T90, TST, and sleep efficiency had no significant correlations with the FOSQ-10 scores (*p* > 0.05). Moderate positive correlations were found between the PCS-12, MCS-12 and FOSQ-10 Total, General Productivity, Activity Level, and Sexual Relationship subscale scores, while weak positive correlations were found between Vigilance and PCS-12 and MCS-12. Moreover, the FOSQ-10 Social Outcomes subscale had a moderate positive correlation with MCS-12 but a weak positive correlation with PCS-12. There were strong negative correlations between the BDI and FOSQ-10 Total, General Productivity, Activity Level, Vigilance, and the Social Outcomes subscale scores, but a moderate negative correlation with the Sexual Relationship subscale score. The BAI score had moderate negative correlations with General Productivity, Vigilance, Social Outcomes, and Sexual Relationship subscale scores, while having strong negative correlations with the FOSQ-10 Total and Activity Level subscale scores (*p* < 0.001). Additionally, there were no significant relationships between the FOSQ-10 Total score and age (*p* = 0.056) and BMI (*p* = 0.087) in OSA patients.

### 3.5. Multiple Regression Model of the FOSQ-10 Total Score

**Figure 1:**
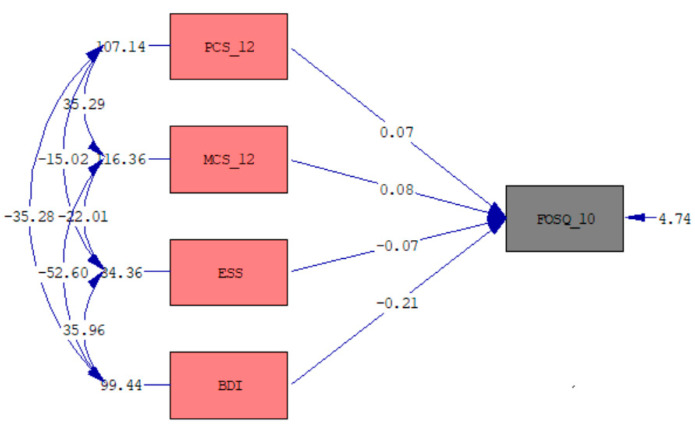
Path diagram of the FOSQ-10 (FOSQ-10: Functional Outcomes of Sleep Questionnaire-10, BDI: Beck Depression Inventory, PCS-12: Physical Component Summary-12, MCS-12: Mental Component Summary-12, ESS: Epworth Sleepiness Scale).

Initially, all sociodemographic, clinical, and other scale scores that were assumed to have an impact on the FOSQ-10 Total score of OSA patients were included in the model. As a result of the modeling, a significant final multiple regression model was achieved. The multiple regression model results and path diagram are given in Table 5 and Figure 1, respectively. The BDI, MCS-12, PCS-12, and ESS scores were found to be simultaneously significantly associated with the FOSQ-10 Total score (F = 182.34, *p* < 0.001). Including these factors, the explained variance in the FOSQ-10 Total score was 69.3%. The MCS-12 and PCS-12 scores had positive effects on the FOSQ-10 Total score, while the BDI and ESS scores had a negative effect. The BDI score was observed to have the greatest impact. For each BDI increase of 1 point, the FOSQ-10 Total score decreased by 0.21 units. When the PCS-12 or MCS-12 scores increased by one point, the FOSQ-10 Total score increased by 0.07 or 0.08 units, respectively. The FOSQ-10 Total score decreased by 0.07 units when the ESS score increased by one point. In Figure 1, the one-way arrows drawn from the PCS-12, MCS-12, ESS, and BDI to the FOSQ-10 indicate the regression coefficients of the multiple regression model. Two-way arrows between independent variables such as PCS-12 and MCS-12 indicate correlated unexplained variances (i.e., 35.29). The short one-way arrows on the variables such as FOSQ-10 and BDI indicate the unexplained variances (i.e., 4.74 and 99.44).

## 4. Discussion

Patients with OSA frequently experience challenges and constraints in executing routine tasks, alongside a decline in their overall quality of life, similar to individuals grappling with various other chronic illnesses such as chronic obstructive pulmonary disease, coronary heart disease, among others. This phenomenon is exacerbated in correspondence with the severity of the OSA condition. The investigation of daily functioning among OSA patients attributed to daytime sleepiness assumes significant importance in comprehending the patients’ state of health and well-being.

The Turkish FOSQ is a 26-item scale that does not include items related to sexual relationships [32]. Since sexual relations are an important aspect of daily functioning, it was preferred to use the shorter FOSQ-10, which included questions regarding sexual activity and reduce participant burden, rather than the longer Turkish FOSQ. As the FOSQ-10 had not been translated into Turkish, this study undertook the process of translating the English FOSQ-10 to Turkish. The analysis of face, content, concurrent, and construct validities demonstrated that the Turkish FOSQ-10 was a valid instrument (Table 2). No cross-cultural adaptation study regarding the content validity of the FOSQ-10 was found in the literature. Previous psychometric evaluations of the FOSQ-10 in OSA patients generally performed analyses such as EFA and correlation analysis for construct validity; however, CFA was not applied. In this study, after a two-factor model was obtained with EFA (total explained variance 58.1%), the construct validity of the FOSQ model was examined by CFA. We obtained comparable results to the study by Rey de Castro et al. [10] conducted in Spain that obtained a two-factor structure (total explained variance 59.9%). The only difference is that while the second factor of their EFA model consisted of two items (Item 3, Item 4), in our study, this factor, “vigilance”, consisted of three items (Item 3, Item 4, Item 10). Item 3 and Item 4 are relevant to the effects of excessive daytime sleepiness or tiredness on driving short and long distances, respectively. The additional Item 10 is related to the effect of excessive daytime sleepiness or tiredness on sexual relationships. We believe the item differences reflect cultural differences between Turkey and Spain. Another factor, “daily activities”, consisted of items (Item 1, Item 2, Items 5–9) that address the effects of excessive daytime sleepiness or tiredness on concentrating, remembering, visiting their home, relationships affected, watching movies, activities in the morning, and activities in the evening of individuals. We found a significant moderate positive correlation between “daily activities” and “vigilance”. The construct validity of the FOSQ model was determined by CFA (χ^2^ = 46.87, df = 130, *p* = 0.026). When this model and the model fit indices were evaluated together (Table 2), this model showed good fit (χ^2^/df < 2, RMSEA < 0.05, SRMR < 0.05, CFI > 0.97, NNFI > 0.97, GFI > 0.95, AGFI > 0.90) [27]. The FOSQ-10 was reliable at a good level (Cronbach’s alpha = 0.82, McDonald’s omega = 0.89, ICC = 0.90) in this study [26,31]. Similar results for the FOSQ-10 were also obtained Chasens et al. [8] (Cronbach’s alpha = 0.87), Rahavi-Ezabadi et al. [14] (Cronbach’s alpha = 0.85, ICC = 0.92), and Rey de Castro et al. [10] (Cronbach alpha = 0.83). As a result, the Turkish translation of the FOSQ-10 was found to be a valid and reliable tool.

The FOSQ-10 Total scores of the moderate and severe OSA patients were lower compared to the negative OSA group. These significant differences were obtained for the Activity Level and Social Outcomes subscales scores of the FOSQ-10. Moreover, the Social Outcomes subscale score of the mild OSA patients was significantly lower than that of the negative OSA group. However, the FOSQ-10 scores did not differ from each other according to OSA severity. There have been differing findings regarding the association between the FOSQ-10 scores and disease severity. For example, Dashzeveg et al. [15] obtained a significant difference in the FOSQ Total score among OSA category, but not among OSA severity. Rahavi-Ezabadi et al. [14] found that there was no significant difference in the FOSQ-10 scores of OSA patients with mild, moderate or severe OSA. Rey de Castro et al. [10] found significant differences in the FOSQ Total score according to OSA severity, while they did not find significant difference in the FOSQ Total score between OSA and non-OSA groups. Banhiran et al. [12], Han et al. [13] (using the Korean FOSQ with 24 items), and Che-Morales and Carrillo-Alduenda [16] also found no significant differences in the FOSQ scores among OSA severity. Contrary to these data, Rey de Castro et al. [10] found significant differences in the FOSQ Total score according to OSA severity while they did not find significant difference in the FOSQ Total score between OSA and non-OSA groups. Additionally, Banhiran et al. [12] and Vidal et al. [33] obtained statistically significant differences in the FOSQ scores between non-OSA/healthy volunteers and OSA patients. 

There were no significant differences between OSA categories in terms of the PCS-12 and MCS-12 subscales scores of the SF-12 in this study. Han et al. [13] found that there was no difference in the quality of life (SF-36) total score according to OSA severity. Silva et al. [9] identified a significant difference in the PCS subscale score of the SF-36 among OSA categories, while they did not find a significant difference in the MCS subscale score of the SF-36 among OSA categories.

Unlike researchers who found that untreated severe OSA patients had decreased quality of life and daily functioning [9,10], we found that, similar to some other studies [12,13,14,15,16], sleep-related quality of life and daily functioning in OSA patients did not differ according to OSA severity.

REM sleep time and the percentages of total sleep time in each sleep stage, excluding total sleep time and efficiency, were significantly different according to the OSA category in this study. Also, similar to the findings of some researchers [12,15,34,35], no significant correlation was found between sleep parameters and the FOSQ Total score in OSA patients. 

In this study, all individuals had moderate depression (20.62 ± 9.7) and anxiety (24.64 ± 9.94) according to the BDI and BAI. The anxiety levels of mild and moderate OSA patients were moderate, while the anxiety level of severe OSA patients was severe. The BDI and BAI scores of severe OSA patients were higher than those of the mild and negative OSA groups. Additionally, the BAI score of moderate OSA patients was higher than that of the negative OSA group. Hong et al. [36] found that the anxiety and depression scores (self-rating depression and anxiety scales) in the moderate–severe OSA patients were significantly higher than those in no-or-mild OSA patients. However, Han et al. [13] and Che-Morales and Carrillo-Alduenda [16] identified that there was no difference in the BDI total score according to OSA severity. 

Daily functioning was positively associated with quality of life, while it was inversely associated with depression, anxiety, and daytime sleepiness in OSA patients in this study. Some researchers obtained results similar to those of this study in terms of the relationships between functional outcomes of sleep and quality of life [7,9,13,14,37,38,39], depression [13,16], and daytime sleepiness [12,13,14,16,36,37,38]. 

In this study, the multiple regression model showed that the BDI, PCS-12, MCS-12, and ESS significantly affected daily functioning in OSA patients. Che-Morales and Carrillo-Alduenda [16] likewise found that the presence of excessive daytime sleepiness (ESS > 10) and depression (BDI > 10) simultaneously significantly affected daily functioning in OSA patients.

The limitations of this study include the lack of a healthy control group without symptoms, such as snoring and daytime sleepiness, which are the most common symptoms, despite the negative OSA group in the study; the fact that the study was conducted in a single center; and that the individuals in the study were middle-aged and had some chronic diseases.

## 5. Conclusions

Daily functioning was more impaired in OSA patients with moderate and severe disease compared to those without OSA. Higher daily functioning in OSA patients contributed to better quality of life but was adversely affected by depression, anxiety, and daytime sleepiness. We suggest that useful tools, such as the FOSQ-10, be employed as a component of the management strategy for OSA to document treatment effectiveness. Additionally, healthcare institutions should offer psychological support to individuals with OSA.

## Figures and Tables

**Table 1:** The sociodemographic and clinical features of individuals according to OSA category.

		Mild OSA (*n* = 83)	Moderate OSA (*n* = 82)	Severe OSA (*n* = 163)	Negative OSA (*n* = 33)	*p*
Age (years) ^&^		46.42 ± 11.85	47.76 ± 10.02	49.42 ± 10.86	44.85 ± 11.91	0.069
Gender *	Male	59 (71.1%)	55 (67.1%)	115 (70.6%)	19 (57.6%)	0.479
	Female	24 (28.9%)	27 (32.9%)	48 (29.4%)	14 (42.4%)	
BMI (kg/m^2^) ^&^		30.37 ± 6.29	32.02 ± 6.32	34.85 ± 6.33	30.27 ± 7.05	<0.001
AHI (events/h) ^#^		9.80 [4.90]	21.20 [6.75]	49 [33.5]	3 [2.55]	<0.001
T90 % ^#^		2.5 [12.2]	15.1 [44.6]	70.5 [136.2]	0 [0.7]	<0.001
TST (min) ^&^		377 ± 45.4	378 ± 46.7	377.6 ± 54.2	377.9 ± 50.5	0.999
SE (%) ^&^		83.1 ± 10.4	84.4 ± 10	83.2 ± 11.7	82 ± 11.2	0.733
REM (%TST) ^&^		13.6 ± 6.8	13.3 ± 6.6	11.1 ± 7.1	14.6 ± 3.9	0.003
N1(%TST) ^&^		9.9 ± 7.3	11.7 ± 8.9	13.2 ± 9.2	12.5 ± 10	0.048
N2 (%TST) ^&^		49.9 ± 10.9	49.4 ± 9.7	56.3 ± 14.1	46.8 ± 10.5	<0.001
N3 (%TST) ^&^		26.6 ± 12.1	25.6 ± 11.9	19.4 ± 12.4	25.4 ± 9.8	<0.001
ESS ^&^		9.13 ± 6.12	10.72 ± 5.41	10.01 ± 5.97	7.61 ± 5.03	0.027
Chronic diseases						
Hypertension (+) *		41 (49.4%)	41 (50%)	88 (54.3%)	10 (30.3%)	0.096
DM (+) *		19 (22.9%)	18 (22%)	45 (27.6%)	4 (12.1%)	0.264
Lung disease (+) *		9 (10.8%)	12 (14.6%)	27 (16.6%)	3 (9.1%)	0.523
Heart disease (+) *		12 (14.5%)	16 (19.5%)	32 (19.6%)	3 (9.1%)	0.408
Neurological disease (+) *		3 (3.6%)	5 (6.1%)	12 (7.4%)	2 (6.1%)	0.715
Chronic diseases (+) *		46 (55.4%)	54 (65.9%)	109 (66.9%)	15 (45.5%)	0.057

* n (%), OSA: Obstructive Sleep Apnea, ^#^ Median [Interquartile Range], ^&^ Mean ±Standard Deviation, BMI: Body Mass Index, AHI: Apnea-Hypopnea Index, T90: Sleep time with oxygen saturation below 90%, TST: Total sleep time, SE: Sleep efficiency, min: minute, REM: Rapid Eye Movement, N1: Stage 1 of None-REM sleep, N2: Stage 2 of None-REM sleep 2, N3: Stage 3 of None-REM sleep, ESS: Epworth Sleepiness Scale, DM: Diabetes Mellitus.

**Table 2:** The psychometric properties of the Turkish FOSQ-10.

Agreement, Validity, and Reliability	Results and Criteria	Decision
Translation ^λ^	Kendall’s W concordance = 100%, ICC = 0.98	Good agreement
Face validity *	1.7 ≤ the impact score of each item ≤ 4.1 ⇒ All impact score ≥ 1.5	Valid
Content validity ^κ^	0.88 ≤ Item-CVI ≤ 1.00 ⇒ Item-CVI ≥ 0.78 Overall average CVI = 0.98 ≥ 0.90 Universal CVI = 0.88	Valid
Concurrent validity	r = −0.56 *p* < 0.001 for the ESS and FOSQ-10 Total r = 0.47 *p* < 0.001 for the PCS-12 and FOSQ-10 Total r = 0.58 *p* < 0.001 for the MCS-12 and FOSQ-10 Total	Valid
Construct validity ^#£^	Minres EFA Kaiser–Meyer–Olkin test = 0.81 Bartlett’s sphericity test χ^2^ = 1347.3 *p* < 0.001 The total explained variance rate of the two-factor model = 58.1%. 0.50 ≤ Factor loadings ≤ 0.93	Valid
	CFA for two-factor model χ^2^ = 46.87, *p* = 0.026, df = 30, χ^2^/df = 1.56 < 2, RMSEA = 0.041 < 0.05, SRMR = 0.038 < 0.05, CFI = 0.99 > 0.97, NNFI = 0.99 > 0.97, GFI = 0.97 > 0.95, AGFI = 0.95 > 0.90 0.49 ≤ Standardized Factor loadings ≤ 0.75	Valid Good fit
Reliability ^ϒ¥^	Cronbach’s alpha = 0.82 > 0.80, McDonald’s omega = 0.89 > 0.80, ICC = 0.90 > 0.75	Good reliability

CVI: Content Validity Index, ESS: Epworth Sleepiness Scale, FOSQ-10: Functional Outcomes of Sleep Questionnaire-10, PCS-12: Physical Component Summary-12, MCS-12: Mental Component Summary-12, EFA: Explanatory Factor Analysis, CFA: Confirmatory Factor Analysis, df: Degree of Freedom, RMSEA: Root Mean Square Error of Approximation, SRMR: Standardized Root Mean Residual, CFI: Comparative Fit Index, NNFI: Non-Normed Fit Index, GFI: Goodness-of Fit Index, AGFI: Adjusted Goodness-of-Fit Index, ICC: Intra Class Correlation, ^λ^ Field (2005) [26], * Lacasse et al. (2002) [25], ^κ^ Almanasreh et al. (2019) [30], ^#^ Cangur and Ercan (2015) [27], ^£^ Hahs-Vaughn (2016) [28], ^ϒ^ Koo and Li (2016) [29], ^¥^ Feißt et al. (2019) [31].

**Table 3:** The levels of functional outcomes, quality of life, depression, and anxiety of individuals according to OSA category.

	Mild OSA (*n* = 83) Mean ± SD	Moderate OSA (*n* = 82) Mean ± SD	Severe OSA (*n* = 163) Mean ± SD	Negative OSA (*n* = 33) Mean ± SD	*p*
*FOSQ-10*					
FOSQ-10 Total	13.9 ± 3.95	13 ± 3.61	13.68 ± 4.07	15.27 ± 2.75	0.040
General Productivity	2.71 ± 0.96	2.65 ± 0.91	2.68 ± 0.93	2.91 ± 0.8	0.561
Activity Level	2.72 ± 0.88	2.41 ± 0.81	2.62 ± 0.86	2.98 ± 0.62	0.007
Vigilance	2.7 ± 0.96	2.61 ± 0.87	2.65 ± 1	3.08 ± 0.61	0.091
Social Outcomes	2.89 ± 1	2.72 ± 1.08	2.98 ± 1.07	3.36 ± 0.6	0.022
Sexual Relationship	2.8 ± 1.05	2.63 ± 1.07	2.63 ± 1.09	2.96 ± 1.16	0.401
Quality of Life (SF-12)					
PCS-12	41.08 ± 9.85	39.93 ± 10.33	39.49 ± 10.63	41.56 ± 9.69	0.576
MCS-12	41.16 ± 10.1	42.01 ± 10.68	42.66 ± 11.2	42.62 ± 10.5	0.768
Depression and Anxious					
BDI	18.30 ± 6.92	20.12 ± 8.92	22.64 ± 11.40	17.70 ± 5.75	0.002
BAI	22.59 ± 6.45	24.23 ± 8.84	26.79 ± 12.02	20.24 ± 4.62	<0.001

SD: Standard Deviation, OSA: Obstructive Sleep Apnea, FOSQ-10: Functional Outcomes of Sleep Questionnaire-10, SF-12: Medical Outcome Survey Short Form 12, PCS-12: Physical Component Summary-12, MCS-12: Mental Component Summary-12, BDI: Beck Depression Inventory; BAI: Beck Anxiety Inventory.

**Table 4:** The correlations between functional outcomes and clinical measures, quality of life, depression, and anxiety levels of OSA patients (*n* = 328).

		FOSQ-10 Total	General Productivity	Activity Level	Vigilance	Social Outcomes	Sexual Relationship
ESS	r	−0.56	−0.45	−0.50	−0.55	−0.42	−0.42
	*p*	<0.001	<0.001	<0.001	<0.001	<0.001	<0.001
AHI	r	0.01	0.03	−0.01	−0.02	0.08	−0.03
	*p*	0.806	0.641	0.819	0.788	0.189	0.666
T90	r	0.04	0.06	0.04	0.06	0.07	−0.04
	*p*	0.448	0.302	0.523	0.316	0.245	0.504
TST (min)	r	−0.08	−0.07	−0.07	−0.05	−0.08	−0.10
	*p*	0.133	0.214	0.190	0.425	0.164	0.105
SE (%)	r	−0.09	−0.11	−0.09	−0.04	−0.06	−0.09
	*p*	0.107	0.056	0.117	0.533	0.298	0.163
PCS-12	r	0.47	0.40	0.45	0.38	0.33	0.47
	*p*	<0.001	<0.001	<0.001	<0.001	<0.001	<0.001
MCS-12	r	0.58	0.56	0.53	0.37	0.45	0.49
	*p*	<0.001	<0.001	<0.001	<0.001	<0.001	<0.001
BDI	r	−0.77	−0.65	−0.69	−0.63	−0.61	−0.59
	*p*	<0.001	<0.001	<0.001	<0.001	<0.001	<0.001
BAI	r	−0.70	−0.59	−0.62	−0.58	−0.53	−0.52
	*p*	<0.001	<0.001	<0.001	<0.001	<0.001	<0.001

FOSQ-10: Functional Outcomes of Sleep Questionnaire-10, ESS: Epworth Sleepiness Scale, AHI: Apnea-Hypopnea Index, T90: Sleep time with oxygen saturation below 90%, TST: Total sleep time, SE: Sleep efficiency, PCS-12: Physical Component Summary-12, MCS-12: Mental Component Summary-12, BDI: Beck Depression Inventory; BAI: Beck Anxiety Inventory.

**Table 5:** Multiple regression model for the FOSQ-10 total score of OSA patients (*n* = 328).

	B	Std. Error	*p*	95.0% Confidence Interval for B	
				Lower Bound	Upper Bound
Constant	12.36	0.89	<0.001	10.61	14.11
BDI	−0.21	0.02	<0.001	−0.24	−0.19
PCS-12	0.07	0.01	<0.001	0.05	0.10
MCS-12	0.08	0.01	<0.001	0.05	0.11
ESS	−0.07	0.03	0.008	−0.12	−0.02
Model Significance	F = 182.344, *p* < 0.001, R^2^ = 0.693				

B: Regression coefficient, Std: Standard, BDI: Beck Depression Inventory, PCS-12: Physical Component Summary-12, MCS-12: Mental Component Summary-12, ESS: Epworth Sleepiness Scale.

## Data Availability

Data will be available upon reasonable request.
